# Trauma Experience Among Women Who Have Substance Use Disorders and are Homeless or Near Homeless

**DOI:** 10.1007/s10597-023-01162-6

**Published:** 2023-07-18

**Authors:** Alison Greene, Josephine D. Korchmaros, Franziska Frank

**Affiliations:** 1 University of Arizona, Southwest Institute for Research on Women, 925 N Tyndall Avenue, P.O. Box 210438, Tucson, AZ 85721-0438 USA; 2 School of Public Health-Bloomington, Indiana University, 1025 East 7th Street, Bloomington, IN 47405-7109 USA; 3 School of Sociology, University of Arizona, 1145 E. South Campus Drive, Tucson, AZ 85721-0438 USA

**Keywords:** Trauma, Substance use, Homelessness, Mental health, Treatment

## Abstract

Women with substance use disorders (SUDs) who are homeless or near homeless have high rates of mental health, behavioral health, and SUD treatment needs. To effectively respond to these needs, it is critical to understand the population-specific trauma experiences of these women. This descriptive study examined the extent and nature of trauma experience among women who have an SUD and are homeless or near homeless. Results (n = 851 women) indicated high rates of trauma experience. All participants (100%) reported at least one type of trauma experience in their lifetime, with the majority (75.3%) having experienced five to seven of the seven types of trauma experiences assessed. Participants reported high levels of emotional severity related to the majority of traumatic events experienced. The pervasiveness of the trauma experiences and the related emotional impact among women with SUDs who are homeless or near homeless reinforce the necessity for trauma-informed care in treatment settings.

## Introduction

Best practices dictate that treatment services are responsive to population needs (Clawson et al., 2008; Substance Abuse and Mental Health Administration [SAMHSA], 2021a). The specific population of women with substance use disorders (SUDs) who are homeless or near homeless have high rates of mental health, behavioral health, and SUD treatment needs (Polcin, 2016; Upshur et al., 2017). Thus, consideration of trauma history is critical when providing services to women with SUDs as it can significantly impact their treatment experiences (SAMHSA, 2014). The clinical care field has evolved to value trauma-informed care and has done so across mental health and SUD treatment systems, which are commonly, though not necessarily, referred to under the broader label of behavioral health.^1^ However, there remains limitations to practitioner understanding of specific population needs related to trauma history. More information regarding the extent and nature of trauma-related issues among women with SUDs who are homeless or near homeless will potentially improve delivery of trauma-informed care and increase the positive impact of treatment services (Hales et al., 2019; Palmieri et al., 2021).

### Women, SUDs, and Mental Health

SUDs are prevalent among women in the United States. In 2019, 7.2 million women ages 18 and older had an SUD, which is approximately 5.6% of all women in the U.S. (SAMHSA, 2020). Women are at a higher risk than men for developing a co-occurring disorder (Graham, 2016), which is the coexistence of both an SUD and a mental illness (SAMHSA, 2022). According to the National Survey on Drug Use and Health (SAMHSA, 2020), in 2019 approximately 4.6 million women (63.9% of those with SUD) had co-occurring SUD and mental illness. Having an SUD is also associated with past year suicidal thoughts (22.5% of women with SUD vs. 4.1% of general population of women), making a plan for suicide (7.7% of women with SUD vs. 1.3% of general population of women), and attempting suicide (3.2% of women with SUD vs. 0.5% of general population of women) (SAMHSA, 2020). Given the prevalence among women of co-occurring SUD and mental illness, clinicians and researchers need to attend more to these overlapping needs as an important approach in treatment (SAMHSA, 2014).

### Homelessness

Homelessness contributes to additional challenges that must be considered in treatment services. The definition of homelessness includes experiences of individuals or families who lack a fixed, regular, and adequate nighttime residence (including living in shelters and transitional housing); who will imminently lose their primary residence; who are fleeing or attempting to flee domestic violence and have no safe, alternative housing; who are trading sex for housing; who are being trafficked; or who are staying with friends, but cannot stay there longer than 14 days (SAMHSA, 2021b; U.S. Department of Housing and Urban Development [HUD], 2019). Those who are near homeless include individuals who are close to fitting the definition of being homeless or whose living situations are tenuous such as those who have received a notice of foreclosure or eviction and don’t have another option for fixed, regular, and adequate nighttime residence. The prevalence of homelessness is difficult to measure, yet it is estimated that in 2020 there were over 580,000 people in the U.S. who were homeless, 38.5% of whom were women (HUD, 2020)^2^. The evidence is mixed on whether causal links exist between homelessness and substance misuse^3^ (McVicar et al., 2015), and if so, in which direction (Allgood & Warren, 2003; Early, 2005; Johnson et al., 1997). However, homelessness is an issue for those who have an SUD, and substance misuse is an issue for those who are homeless. Even without prior or current experiences of homelessness, having an SUD increases risk for first-time homelessness (Thompson et al., 2013). SUD rates among women who are homeless are significantly higher as compared to the general population of women (Polcin, 2016), with a recent study finding a rate of 12 times higher (Upshur et al., 2017). Additionally, in 2020, 17.0% of homeless individuals had chronic substance misuse and 20.8% had severe mental illness (HUD, 2020). Finally, individuals experiencing homelessness are disproportionately victims of violence-related mortality, including homicide and suicide, as compared to the broader population (Kleinman et al., 2023).

### Women and Trauma

Women’s experiences of trauma and homelessness are closely interconnected (Milaney et al., 2020). Previous studies have found that women who are homeless are a highly traumatized group and have more significant histories of traumatic stress and interpersonal violence than women who are housed (McHugo et al., 2005; Pavao et al., 2007; Wenzel et al., 2000). Trauma experiences such as intimate partner violence are often pathways to forced displacement and homelessness (Pavao et al., 2007; U.S. Conference of Mayors, 2016). Additionally, while homeless, women are at higher risk for further trauma as they are particularly vulnerable to victimization such as sexual and/or physical assault and witness to violence towards others (Sikich et al., 2008; Wenzel et al., 2000).

Histories of trauma are also very high among women with SUD and those who misuse substances (McHugo et al., 2005; Torchalla et al., 2014). It is estimated that 80–90% of individuals (women and men) who misuse substances and are treatment-seeking or in treatment have lifetime histories of victimization and other traumatic events (Brown et al., 1999; McHugo et al., 2005; Reynolds et al., 2005). Women’s experiences of repeated, chronic, or multiple traumas can result in pronounced symptoms and consequences, including SUD, mental health issues, and other health problems that can impact engagement in treatment (Brown et al., 1999; McHugo et al., 2005; Reynolds et al., 2005; Torchalla et al., 2014). Additionally, among women with SUD, having a trauma history increases the likelihood of having more severe and complex clinical presentations, having difficulty with treatment engagement and retention, and having generally worse SUD outcomes (Brady et al., 1998). Reducing trauma symptoms during treatment can improve women’s ability to manage their substance misuse post-treatment (Hien et al., 2010; López et al., 2015; SAMHSA, 2014). Thus, consideration of women’s experiences with trauma is necessary for effective SUD treatment, particularly among women who are homeless or near homeless and have SUD. In addition, because of the common co-occurrence of SUD and mental illness, consideration of women’s experiences with trauma is necessary for effective mental health treatment of women who have SUD.

### Trauma-Informed Care

Given the significance of SUD and mental illness, the impact of lifetime trauma experiences needs to be integrated into clinical approaches through trauma-informed care and utilized when working with women who are at risk for or are currently experiencing homelessness. Trauma-informed care is a set of approaches used in clinical practice that translate the science of how trauma is processed in the brain to inform service provision that addresses the symptoms of the trauma (SAMHSA, 2014; Tkach, 2018). Trauma-informed care prioritizes an individual’s life experiences in delivering effective care (Menschner & Maul, 2016) and its use is appropriate for a range of practitioners across diverse clinical and treatment settings (Tkach, 2018).

### Current Study

The purpose of this descriptive study was to examine the rates of trauma experience among women who have an SUD or misuse drugs and are homeless or near homeless. To effectively deliver trauma-informed care and address the needs, experiences, and related symptoms of individuals, it is critical to understand the trauma of the population. Understanding the type(s) and extent of trauma can inform clinical approach across treatment settings and provide opportunities to tailor treatments to the specific population. This paper focuses on the extent and nature of trauma experiences among women who have an SUD or misuse drugs and are homeless or near homeless.

## Methods

### Participants

A total of 851 women participated in this study. At the time of enrollment, participants were 18 years of age and older, were homeless or near homeless, and were either receiving SUD treatment or were not in treatment but were currently misusing substances.

### Measures

#### Demographics and Participant Characteristics

The researchers queried ethnicity (Hispanic: yes/no), race (Black or African American; Asian; Native Hawaiian or other Pacific Islander; Alaska Native; White; American Indian; when more than one category was endorsed, researchers coded response as ‘Multiracial’), age, and living situation (shelter; street/outdoors; institution [hospital; nursing home; jail/prison]; housed in an apartment, room, or house they own/rent; housed in someone else’s apartment, room, or house; college residence; halfway house or boarding house; other) using the Government Performance Results Act Client Outcome Measures for Discretionary Programs (GPRA) Tool (SAMHSA, 2010). For the purposes of this study, researchers also asked an open-ended question about current marital status and then coded responses into categories (single; married; divorced; separated; widowed; living with partner long enough to be considered in a common law marriage; living with sexual partner; in some other relationship status).

#### Trauma Experience

The researchers measured trauma and environmental stress with the Environmental Stress Inventory (ESI; Stevens and Murphy, 1998). ESI assesses trauma experiences in childhood and throughout the lifespan of the participants. The ESI includes questions about 65 specific traumatic events. For the purposes of the present study, we analyzed the data from the 42 specific traumatic events spanning the following seven types of traumatic experiences commonly focused on in the fields of trauma, behavioral health, and mental health (Table 1): (1) *Sexual Abuse and Assault* (4 specific events); (2) *Physical Abuse and Assault* (2 specific events); (3) *Non-interpersonal Threat to Physical Health* (5 specific events); (4) *Forced Displacement* (4 specific events); (5) *Traumatic Grief and Separation from Children* (8 specific events), *Drug Partners* (14 specific events), *and Family* (3 specific events); (6) *Exposure to Community Violence* (1 specific event); and (7) *Justice Involvement* (1 specific event). Of the 23 items that were excluded, 14 were excluded because they did not fit the trauma categories of focus (e.g., you gained a lot of weight, you used drugs/alcohol with a younger sibling, and you had a firearm in your house) and 9 overlapped significantly with items already included by either being more general or more specific (e.g., divorce of your parents and death of a child close to you).

**Table 1:** Trauma experienced during lifetime and the past 30 days and experienced severity

	% experienced during life time (n = 851)	% experienced during past 30 days (n = 851)	Mean level of upset (SD)
1. Sexual Abuse and Assault	75.6	23.0	
Being raped	54.1	3.6	4.84 (0.63)
Fear of sexual advances	53.1	21.3	4.18 (1.11)
A person in an authority position made sexual advances towards you	26.4	2.0	3.85 (1.41)
Your husband /significant other pimped you off or set you up to have sex with someone	11.2	1.6	4.03 (1.34)
2. Physical Abuse and Assault	75.9	33.3	
You feared that someone might physically hurt you	67.1	31.1	4.46 (0.89)
You physically hurt someone in your family	34.8	4.7	3.94 (1.35)
3. Non-interpersonal Threat to One’s Physical Health	83.2	32.3	
You thought about hurting or killing yourself	52.2	19.6	4.28 (1.27)
You had a serious accident or illness	49.7	10.6	4.44 (0.94)
You were told that you have a sexually transmitted infection (STI)	44.1	3.8	4.54 (0.89)
You were told that you have some kind of physical/medical condition that inhibits you from normal activity	24.9	9.9	3.87 (1.39)
You were told that you have HIV/AIDS	0.9	0.0	4.88 (0.35)
4. Forced Displacement	85.0	43.2	
You didn’t have a stable place to live	71.3	37.4	4.53 (0.87)
Your husband/significant other/family kicked you out of their home	43.2	10.8	4.50 (0.98)
You lost your home through fire/financial, etc.	40.3	7.8	4.63 (0.81)
You went to prison	16.8	1.5	4.34 (1.14)
5. Traumatic Grief/Separation	99.9	37.3	
A. Children	81.1	16.2	
Death of your child	8.7	0.2	5.00 (0.00)
You gave child(ren) up for adoption	10.5	0.8	4.48 (1.09)
Your husband/significant other /family took your children away from you	24.2	3.6	4.72 (0.90)
You lost children to authorities	27.6	6.0	4.93 (0.51)
You had a miscarriage	40.3	1.1	4.18 (1.24)
You had an abortion	39.0	0.7	4.13 (1.25)
Your child(ren) were removed by authorities	41.5	10.9	4.99 (0.22)
You left your child(ren) as collateral for drugs	0.4	0.1	5.00 (0.00)
B. Immediate Family	89.4	16.6	
Mother died of drug related causes (drug overdose, violence)	3.8	0.0	4.62 (1.19)
You never knew your mother	6.2	n/a	2.49 (1.79)
Siblings died of drug related causes (drug overdose, violence)	7.3	0.5	4.76 (0.74)
Father died of drug related causes (drug overdose, violence)	7.9	0.4	4.20 (1.50)
You were adopted	10.0	n/a	2.51 (1.69)
Mother died of sickness	12.2	0.4	4.67 (0.97)
You never knew your father	16.3	n/a	3.10 (1.76)
Father died of sickness	21.3	0.5	4.49 (1.16)
Murder of a close family member	22.0	0.5	4.71 (0.76)
You were abandoned by your parents	36.4	3.1	4.53 (1.02)
Separation of your parents	53.6	0.9	3.62 (1.59)
Father went to jail (more than a year)	16.3	1.2	3.10 (1.80)
Mother went to jail (more than a year)	6.0	0.2	3.63 (1.62)
Siblings went to jail (more than a year)	29.3	4.0	3.72 (1.48)
C. Friends and Drug Partners	75.2	14.0	
Your husband/significant other left you	49.0	8.6	4.50 (0.99)
Drug partners death (drug overdose, being shot, etc.)	32.5	3.8	4.58 (0.84)
Friends that died violently	49.2	3.9	4.68 (0.67)
6. Victim of/Witness to Community Violence	32.9	3.4	
You or your friends were shot at	32.9	3.4	4.12 (1.34)
7. Justice Involvement	82.6	31.0	
You got in trouble with the law	82.6	31.0	4.31 (1.13)

Study participant experience with each of the 42 specific traumatic events was assessed with two questions of interest for the present study: *“Have you ever experienced X during your lifetime? (Yes/No)”*; and *“Have you experienced X in the past 30 days? (Yes/No).”* For the purposes of the present study, responses to these questions provided a comprehensive picture of a participant’s trauma experience in terms of which specific traumatic events were experienced ever and recently. It is important to note that the ESI categories include trauma caused by a broad variety of stressful life events and, as such, go beyond the DSM-V Criterion A types of trauma (American Psychiatric Association, 2013; Pai et al., 2017).

#### Emotional Severity of the Trauma

To assess the experienced emotional severity of the traumatic event, we asked participants to describe their level of upset at the time the event happened. More specifically, for each traumatic event each participant reported experiencing, we asked: *“How upset were you, when X happened to you?”* Response options were on a 5-point scale ranging from “not at all upset” (1) to “extremely upset” (5). We calculated average level of upset reported in relation to each specific trauma event queried. Each mean reflects the mean level of upset reported by the subset of participants who reported experiencing the event. We did not calculate mean level of upset at the type of trauma category level because different participants and different numbers of participants experienced each of the specific events within each type of trauma category and, so, different participants and different numbers of participants reported level of upset to each specific event queried. Therefore, it was not appropriate to calculate mean level of upset at the type of trauma category level.

### Procedure

Study data were collected in one city in the U.S. as part of Mujer Sana ~ Healthy Woman, an HIV, sexually transmitted infections (STI), hepatitis, and tuberculosis health education project for women with substance use issues. Mujer Sana was funded by the Substance Abuse and Mental Health Service Administration (SAMHSA, H79TI14452). To be eligible to participate, women had to be 18 years of age or older at the time of enrollment and either be receiving treatment or services from a residential SUD treatment facility or currently misusing substances, but not receiving treatment. Although being homeless or near homeless was not a specific eligibility criterion for the study, the study researchers aimed to recruit women who were homeless or near homeless as indicated by their current living situation or their lacking stable income adequate to support stable living situations after completing residential treatment. Health educators identified potential participants through street outreach, programs for the homeless, and residential SUD treatment. After screening potential participants for study eligibility, health educators invited the women who met eligibility criteria to be in the study. During the consenting procedure, the health educators explained the health education project and what participants would experience if they enrolled. This explanation included a description of the evaluation component of the project, which included participants completing a baseline assessment prior to participating in the education program and two follow-up data collection sessions at three and six months post-baseline. To eliminate the impact of literacy on completion of the assessment, researchers conducted the assessment in the form of an interview during which researchers read aloud the assessment items and recorded participants’ responses. Researchers conducted the assessment interviews as soon as possible after consent was obtained. Researchers informed participants that their participation was voluntary and choosing not to answer assessment questions or their discontinuing their participation would not impact their receipt of services nor their opportunity to participate in the health education component of Mujer Sana. Participants did not receive a monetary incentive for completing the baseline survey, though they received $20 gift cards for completing each of the follow-up data collection sessions. Data from the baseline assessment were analyzed for the purposes of the present study. All data and procedures related to this study were approved by the Human Subjects Institutional Review Board at the University of Arizona.

## Results

### Demographics and Participant Characteristics

Of the 851 participants, 100% identified as women. Participants ranged in age from 18 to 69 years old with an average age of 31.98 years old. 25% were 18 to 24 years old, 36.4% were 25 to 34 years old, 27.7% were 35 to 44 years old, and 10.5% were 45 to 69 years old. Approximately a quarter of participants (26.6%) identified as Hispanic, with 4.2% who solely identified as Hispanic with no racial identification provided. The majority of participants (60.6%) identified as White, 10.8% identified as American Indian, 6.7% as Black or African American, 6.6% as Multiracial, 10.0% as an “Other” race, and 1.1% did not report their race.

There was diversity in relationship status and living situation reported by the women. Of the 851 participants, 44.8% were single, 18.9% were divorced with another 10.0% separated, 15.1% were married and an additional 3.1% had been living with a partner long enough to be considered in a common law marriage, 5.4% were living with a sexual partner, 2.2% were widowed, and 0.5% had some other relationship status. At the time of enrollment, the majority of participants (76.6%) were living in residential treatment, 8.6% were living in someone else’s house, 7.7% were living in their own house, and 5% were living on the street or in a shelter, halfway house or boarding house. There were 1.1% of participants living in a hotel, and 0.6% in jail, and 0.4% in some other living arrangement. Prospects of stable housing for those in residential treatment after leaving treatment as well as for the other participants were limited as 78.5% of participants were unemployed or laid off.

### Overall Trauma Experiences

**Fig. 1:**
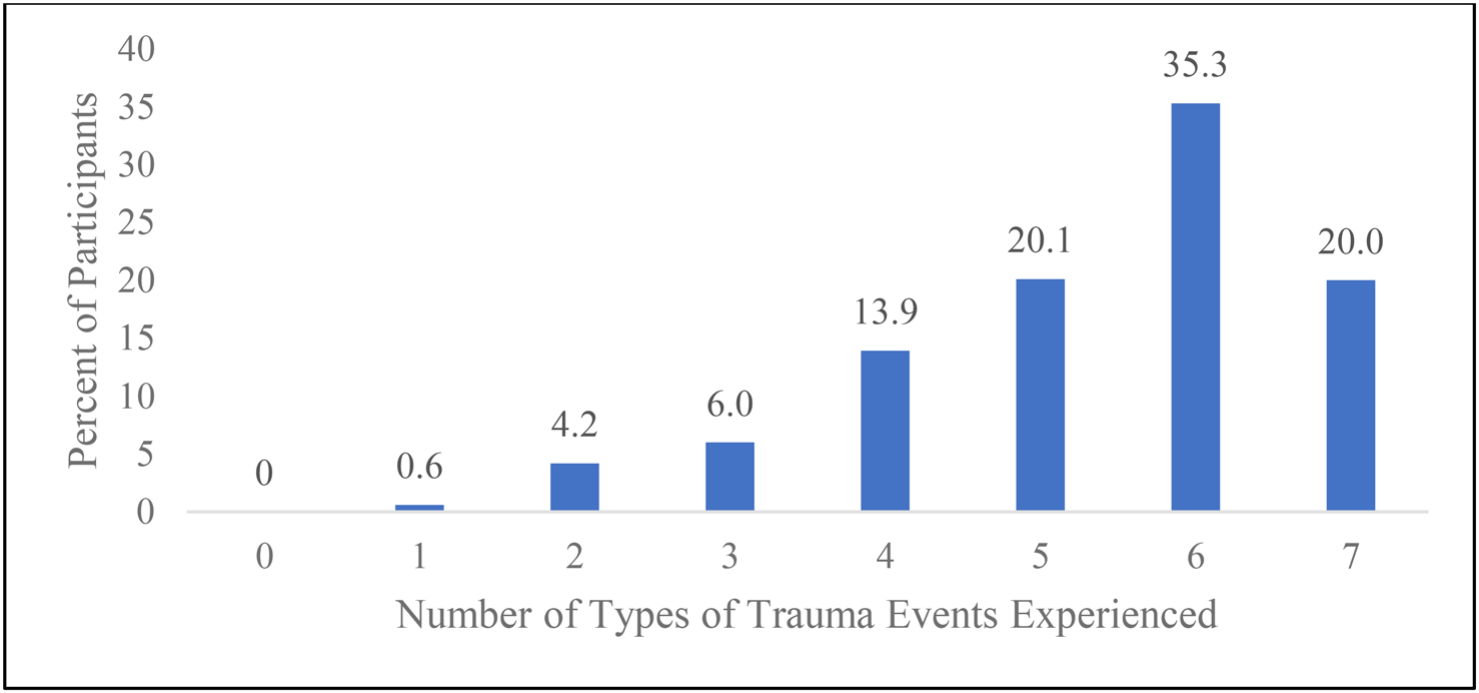
Number of types of trauma events experienced during lifetime

The data indicate high rates of experience with trauma in women who misuse drugs and are homeless or near homeless. Figure 1 shows the prevalence of different types of traumatic experiences experienced by women in our sample based on the seven types of trauma of interest in the current study. The figure shows that every woman experienced at least one type of trauma experience in their lifetime, with the majority (75.3%) having experienced five to seven of the seven different types of trauma experiences assessed. One fifth of the women (20.0%) had experienced all seven types of trauma during their lifetime.

**Fig. 2:**
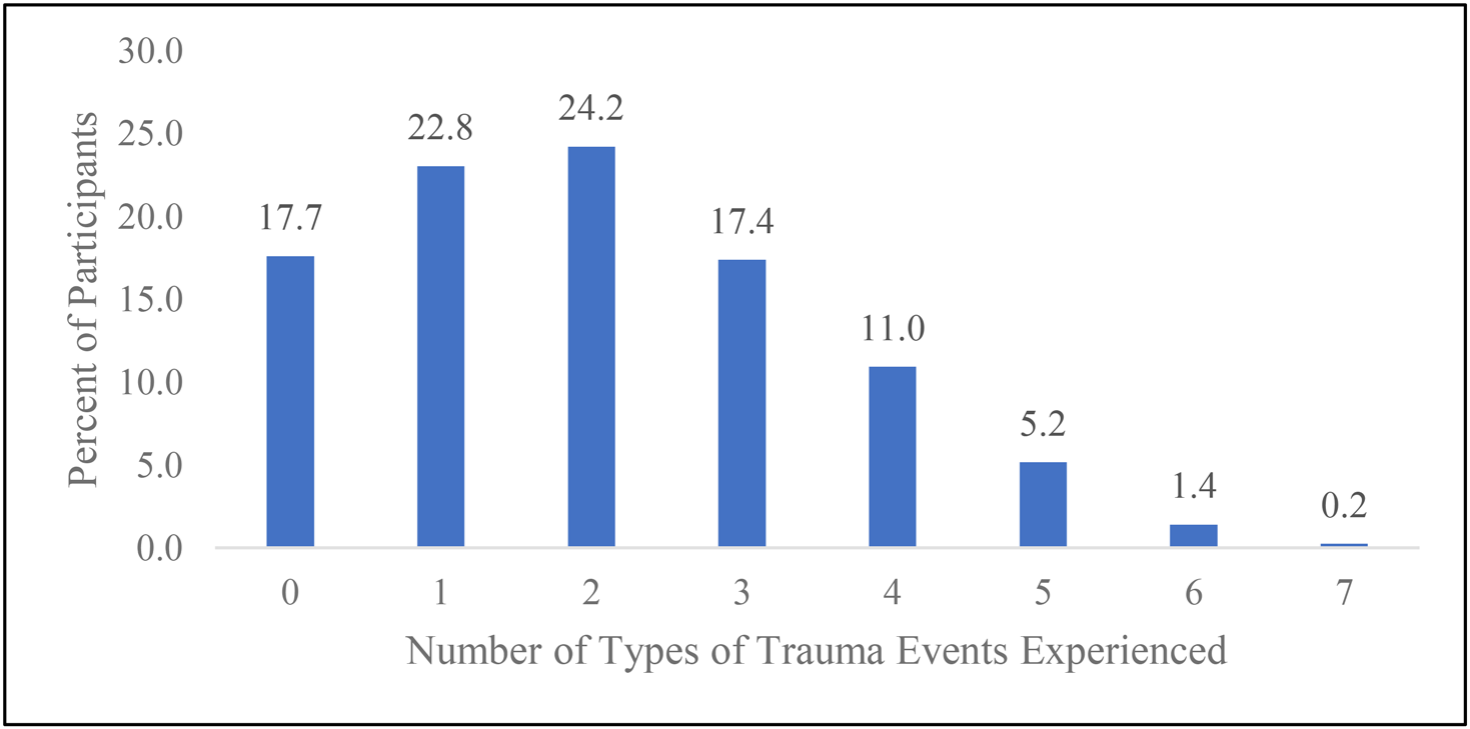
Number of types of trauma events experienced during past 30 days

Figure 2 shows the number of types of trauma experienced recently—during the past 30 days. Although 17.7% reported no recent experiences of any of the seven types of trauma assessed, the majority (82.3%) experienced at least one of the seven types of trauma within the past 30 days. During the past 30 days, most participants were exposed to two or more of the types of trauma (59.5%) and more than a third (35.3%) reported experiencing three or more of the seven types of trauma.

Table 1 shows the prevalence of the seven different trauma types and the specific traumatic event(s) per each type experienced over the women’s lifetime and recently, within the past 30 days. Traumatic grief and separation was the category with the highest endorsed lifetime events as nearly all women (99.9%) reported lifetime experiences, and it was the second highest category with endorsed recent events reported in the past 30 days (37.3%). Forced displacement was the category with the highest endorsed recent events (43.2%), and the second highest endorsed for lifetime events (85.0%). All other categories of trauma experience were relatively high for both lifetime (ranges from 75.6 to 83.2%) and recent (31.0–33.3%) experiences with the exception of community violence (32.9% lifetime; 3.4% past 30 days).

Among the specific 42 traumatic events, the five most commonly reported lifetime experiences among the women were (1) trouble with the law (82.6%); (2) didn’t have a stable place to live (71.3%); (3) feared that someone might physically hurt you (67.1%); (4) being raped (54.1%); and (5) separation of your parents (53.6%). The next five most commonly reported lifetime experiences among the women were (6) fear of sexual advances (53.1%); (7) thought about hurting or killing self (52.2%); (8) had a serious accident or illness (49.7%); (9) friends that died violently (49.2%); and (10) husband/significant other left (49.0%).

The five most commonly reported recent events that occurred in the past 30 days were (1) didn’t have a stable place to live (37.4%); (2) feared that someone might physically hurt you (31.1%); (3) trouble with the law (31.0%); (4) fear of sexual advances (21.3%); and (5) thought about hurting or killing self (19.6%). The next five most commonly reported recent events that occurred in the past 30 days were (6) child(ren) were removed by authorities (such as Child Protective Services) (10.9%); (7) husband/significant other/family kicked you out of their home (10.8%); (8) had a serious accident or illness (10.6%); (9) told you have some kind of physical/medical condition that inhibits you from normal activity (9.9%); and (10) husband/significant other left (8.6%).

Table 1 also shows the reported severity of the 42 traumatic events spanning the seven types of traumatic experiences. In general, women reported high levels of emotional severity related to the majority of traumatic events experienced. Thirty-two traumatic events had average ratings equal to or greater than 4.0, which is ‘considerably’ to ‘extremely upsetting.’ All women who experienced ‘death of your child or you left your child(ren) as collateral for drugs’ rated those events as extremely upsetting (*M* = 5.00). Women rated a third specific event of ‘your child(ren) were removed by authorities (CPS)’ on average at 4.99. These 32 traumatic events spanned all seven trauma types. Only two of the 42 trauma events were, on average, less than ‘moderately upsetting’ at 2.49 (‘you never knew your mother’) and at 2.51 (‘you were adopted’).

### Sexual Abuse and Assault

As shown in Table 1, approximately three quarters (75.6%) of the women experienced sexual abuse or assault in their lifetime and almost a quarter (23.0%) experienced sexual abuse or assault during the past 30 days. More than half of the women reported having been raped (54.1%) or having feared that someone might make sexual advances toward them (53.1%) at least once in their lifetime. The fear of someone in a position of authority making sexual advances appears to be relatively persistent with 26.4% reporting having this fear during their lifetime. Also notable is the rate of sex trafficking experienced, with 11.2% of participants reporting having had this experience in their lifetime. On average, women rated these traumatic experiences as close to ‘considerably’ upsetting to ‘extremely’ upsetting (*M* = 3.85 to 4.84).

### Physical Abuse and Assault

Like sexual abuse and assault, physical violence is very prevalent in the lives of women who have SUDs and are homeless or near homeless. Three quarters of participants (75.9%) experienced physical abuse or assault during their lifetime. About two thirds (67.1%) of the women had feared that someone might physically hurt them at least once during their lifetime, which they reported as being, on average, ‘considerably’ to ‘extremely’ upsetting (*M* = 4.47). Furthermore, the fear of being physically hurt is pervasive in women’s recent lived experience with 31.1% of participants reporting having experienced this fear during the past 30 days. The participants also commonly reported having engaged in violence towards others, with more than a third (34.8%) of the women reporting having physically hurt someone in their own family at least once in their lifetime. On average, they reported this event to be ‘moderately’ to ‘considerably’ upsetting (*M* = 3.94).

### Non-interpersonal Threat to One’s Physical Health

A majority of the participants (83.2%) reported being confronted with non-interpersonal threats to their physical health. More than half (52.2%) reported having had suicidal thoughts during their lifetime, and about a fifth (19.6%) have had such thoughts during the past 30 days. On average, they found these events ‘considerably’ to ‘extremely’ upsetting (*M* = 4.28). A tenth (9.9%) of the participants had been recently diagnosed with a medical condition that inhibits them from normal activity, while nearly a quarter (24.9%) had been confronted with such diagnoses in their lifetime. On average, they found these events to be ‘moderately’ to ‘considerably’ upsetting (*M* = 3.87). During their lifetime, 44.1% of women had been informed that they had contracted a sexually transmitted infection (STI), with 0.9% having been diagnosed with HIV/AIDS, both of which they found ‘considerably’ to ‘extremely’ upsetting (*M* = 4.54 and 4.88, respectively).

### Forced Displacement

The majority (85.0%) of the participants experienced forced displacement in some way at some point in their lives. Seven in ten (71.3%) reported having been confronted with not having a stable place to live and four in ten (43.2%) reported having been kicked out of the homes of their husband, significant other, or family member. At some point during their lifetime, 40.3% of participants lost their home through either fire, financial, or other reason. In addition, 16.8% went to prison at least once in their life, with 1.5% having been incarcerated during the past 30 days. Approximately every third woman (37.4%) did not have a stable place to live during the past 30 days. Women reported feeling ‘considerably’ to ‘extremely’ upset by these traumatic events (*M* = 4.34 to 4.63).

### Traumatic Grief/Separation

Virtually all (99.9%) study participants experienced some type of traumatic grief and separation event, with more than one third (37.3%) having recently experienced some type of traumatic grief and separation event. Eight in ten (81.1%) indicated traumatic grief and separation from their children which across specific related traumatic events resulted in women feeling, on average, ‘considerably’ to ‘extremely’ upset (*M* = 4.13 to 5.00). Four in ten (41.5%) participants reported that their children had been removed by authorities sometime in their life, while one in ten (10.9%) experienced this recently. One in ten (10.5%) participants had given child(ren) up for adoption, while 39.0% had had an abortion, and 40.3% had had a miscarriage at some point in their life. Across all trauma events queried, traumatic grief and separations from children had the most emotional impact with women reporting feeling extremely upset regarding death of their child (*M* = 5.00), having their child(ren) removed by authorities (*M* = 4.99), and having left their child(ren) as collateral for drugs (*M* = 5.00). Even being told that they had HIV/AIDS was not as upsetting as these events related to traumatic grief and separations from children.

Most (89.4%) participants experienced traumatic grief and separation from immediate family (i.e., parents and siblings). Many participants indicated that they were abandoned by their own parents (36.4%), and more than half (53.6%) experienced their parents’ separation. Also of note, is the prevalence of loss due to violence and drug misuse, with some participants having experienced death of a father (7.9%), mother (3.8%), or sibling (7.3%) because of a drug overdose or drug related violence. These experiences resulted in women feeling ‘considerably’ to ‘extremely’ upset (*M* = 4.20, 4.62, and 4.76, respectively). Many women (22.0%) experienced the murder of a close family member which resulted in their feeling ‘considerably’ to ‘extremely’ upset (*M* = 4.71). In addition, a large percentage of participants experienced separation from a sibling (29.3%), father (16.3%), or mother (6.0%) due to family members’ detainment in jail for more than a year, which women reported as ‘moderately’ to ‘considerably’ upsetting (*M* = 3.72, 3.10, and 3.63, respectively).

Many (75.2%) participants experienced traumatic grief through loss of or separation from friends and drug partners. Almost half (49.0%) of participants had been, at least once in their lifetime, left by their husband or significant other, with 8.6% of participants having experienced this loss in the past 30 days. Almost one third (32.5%) of participants indicated that they faced grief through the loss of a drug partner. In addition, almost half (49.2%) of participants reported traumatic grief related to having had friends who died violently. Women reported feeling ‘considerably’ to ‘extremely’ upset (*M* = 4.50, 4.58, and 4.68, respectively) with all of these traumatic events related to friends and drug partners.

### Victim of/Witness to Community Violence

Participants also experienced trauma related to community violence as nearly one third (32.9%) of participants reported that they or their friends were the target of gunfire. Women reported feeling ‘considerably’ to ‘extremely’ upset (*M* = 4.12) by this form of trauma.

### Justice Involvement

Justice involvement was prevalent among participants. The majority (82.6%) of study participants reported having been in trouble with the law at some point during their lives, while about a third (31.0%) had gotten into trouble with the law during the past 30 days. Justice involvement was ‘considerably’ to ‘extremely’ upsetting (*M* = 4.31) to the women impacted. While 82.6% of the women reported justice-involvement, only 16.8% reported a prison stay (as reported in relation to forced displacement).

## Discussion

The prevalence and severity of lifetime and recent trauma experiences among women with SUDs who are homeless or near homeless are alarming. This subpopulation of women reported extensive and impactful occurrences across different types of trauma. All participants had experienced at least one type of trauma, but nearly four-fifths had experienced five or more types and one fifth had experienced all seven assessed types of trauma during their lifetime. Over three quarters of the women reported lifetime experiences of trauma within six of the seven trauma categories.

While experiences of trauma among the general population are not uncommon, the prevalence is much lower as compared to women with SUDs who are homeless or near homeless. A general population survey conducted in over 20 countries found that 70.4% of adults experienced a lifetime traumatic event (Benjet et al., 2016) as compared to 100% of participants in the current study. According to the National Intimate Partner and Sexual Violence Survey, almost one in five women (18.3%) in the general U.S. population experience rape at least once in their lifetime (Smith et al., 2018), yet we found that over half (54.1%) of women with SUDs who are homeless or near homeless in our study reported having been raped. In the general U.S. population in 2020, 4.9% of adults reported having had serious thoughts of suicide in the past year (SAMHSA, 2021c) as compared to 19.6% of women in this study having had suicidal thoughts in the past 30 days, and 52.2% reported having these thoughts over the course of their lifetime. The pervasiveness of the trauma experiences and the related emotional impact among women with SUDs who are homeless or near homeless reinforce the necessity for trauma-informed care in treatment settings.

The trauma experience profile of this subpopulation of women with SUDs who are homeless or near homeless is useful for informing both the approach to treatment services, as well as the clinical experience to best meet the needs of individual women. Across mental health and SUD treatment settings, as well as the broader behavioral health treatment setting, there are opportunities to provide a trauma-informed approach to care and service provision. Descriptions of the trauma histories of specific populations, such as provided by the present study, can be used to direct trauma-informed care to increase the responsiveness of practice to the lived experiences of the populations seeking clinical care. Greene and Korchmaros (2023) provide a detailed description of how trauma-informed care can be developed or adapted to be responsive to known particulars of the trauma histories of women who have SUDs and are homeless are near homeless.

### Limitations

The present study has some limitations. For instance, we did not ask women how often they experienced each specific type of trauma event, but it is important to keep in mind that many of the types of trauma can and are often experienced repeatedly. This, as well as the timing of the data collection as compared to the trauma experience occurrence, may have impacted the participants’ reports of emotional severity. The traumatic event experiences that each participant reported as well as the emotional severity ratings were their current perceptions at the time of data collection and need to be considered within the larger context of their lives. The data were self-reported, which comes with limitations (e.g., individuals may/may not want to share; impact of memory; from one person’s perspective). However, the self-report approach is consistent with our recommendations for clinical care to be tailored and responsive to the specific population needs as defined, interpreted, and understood by the individuals who experience them. Further, the information needed to inform a trauma profile of the population is not consistently reported anywhere. Consequently, relying on self-reported data is a direct and informative approach. Another potential limitation is related to geography and culture. This study was conducted in one city in the Southwestern U.S. and there may be some regional variation, but we do not expect that the variation would be enough to negate the findings. Women with substance misuse issues who are homeless/near homeless are particularly vulnerable to trauma experiences, but some of our findings or the extent of the findings may be particular to region or culture. Finally, our sample only included women experiencing homelessness or near homelessness who also had SUDs or substance misuse; future research focused on comparisons of trauma experience among women experiencing homelessness or near homelessness with and without substance-related issues could result in interesting findings.

## Conclusion

This descriptive study provides a trauma experience profile of women with SUDs who are homeless or near homeless. The results reinforce that this population experiences extensive and emotionally severe trauma over their lifetime. Applying these findings to clinical practice may improve engagement and retention of women with SUDs who are homeless or near homeless in supportive healthcare, improve service provision to this population, and improve service effectiveness.

## Footnotes

1 Behavioral health is related to the actions people take. It is a term often used to include mental health. Although psychological and physiological factors dominate in mental health disorders, the presentation of mental disorders is often behavioral, such as in aggressive tendencies. SUDs are a specific type of mental health disorder. Furthermore, because multiple behavioral health issues, mental health issues, and SUDs often co-occur, the associated treatment systems are often coordinated or integrated in practice. Consequently, the associated clinical care systems are distinct though overlap substantially. For example, in practice, behavioral health treatment can include treatment to address SUDs and/or treatment to address externalizing mental disorders such as aggression because of the specific focus, expertise, and capacity of the provider and the needs of the populations it serves. But it doesn’t necessarily include SUD treatment nor treatment for internalizing mental disorders, such as depression.

2 These data are from the HUD report on homelessness which is based on point-in-time information; readers should be cautioned that there are many limitations in measuring homelessness, including that many people enter and exit homelessness in a given year. For more information on point-in-time count methodology see https://www.hudexchange.info/hdx/guides/pit-hic/.

3 We use “SUD” when there is a verified clinical diagnosis of SUD. We use “substance misuse” to indicate the behavior of misusing substances.
